# XRCC2 as a predictive biomarker for radioresistance in locally advanced rectal cancer patients undergoing preoperative radiotherapy

**DOI:** 10.18632/oncotarget.4975

**Published:** 2015-07-22

**Authors:** Chang-Jiang Qin, Xin-Ming Song, Zhi-Hui Chen, Xue-Qun Ren, Kai-Wu Xu, Hong Jing, Yu-Long He

**Affiliations:** ^1^ Department of Gastrointestinal and Pancreatic Surgery, The First Affiliated Hospital of Sun Yat-sen University, Guangzhou, China; ^2^ Department of Gastrointestinal Surgery, Huaihe Hospital of Hennan University, Kaifeng, China; ^3^ Department of Pathology, Huaihe Hospital of Hennan University, Kaifeng, China

**Keywords:** XRCC2, rectal cancer, preoperative radiotherapy, radioresistance

## Abstract

XRCC2 has been shown to increase the radioresistance of some cancers. Here, XRCC2 expression was investigated as a predictor of preoperative radiotherapy (PRT) treatment response in locally advanced rectal cancer (LARC). XRCC2 was found to be overexpressed in rectal cancer tissues resected from patients who underwent surgery without PRT. In addition, overall survival for LARC patients was improved in XRCC2-negative patients compared with XRCC2-positive patients after treatment with PRT (*P* < 0.001). XRCC2 expression was also associated with an increase in LARC radioresistance. Conversely, *XRCC2*-deficient cancer cells were more sensitive to irradiation *in vitro*, and a higher proportion of these cells underwent cell death induced by G2/M phase arrest and apoptosis. When *XRCC2* was knocked down, the repair of DNA double-strand breaks caused by irradiation was impaired. Therefore, XRCC2 may increases LARC radioresistance by repairing DNA double-strand breaks and preventing cancer cell apoptosis. Moreover, the present data suggest that XRCC2 is a useful predictive biomarker of PRT treatment response in LARC patients. Thus, inhibition of XRCC2 expression or activity represents a potential therapeutic strategy for improving PRT response in LARC patients.

## INTRODUCTION

Rectal cancer is currently one of the most common human malignancies diagnosed. In China, the incidence of rectal cancer is increasing at a rate of 4.2% per year [1]. Preoperative radiotherapy (PRT) is an essential treatment option for locally advanced rectal cancer (LARC) [2-4]. However, its efficacy is highly debated as responses to PRT have been found to vary among individuals [5-7]. Several biomarkers for predicting PRT response in LARC patients have been investigated, although their clinical applications remain unclear [8-11]. Thus, the investigation of novel biomarkers to predict radiosensitivity, as well as the tailoring of treatments to individual patients, are imperative [12, 13].

Ionizing radiation (IR) kills tumor cells mainly by inducing DNA double-strand breaks (DSBs) [14]. The homologous recombination repair (HRR) pathway plays a role in repairing radiation-induced DNA DSBs, and inhibition of this pathway could improve radiosensitivity [15-17]. In the HRR pathway, the protein, X-ray repair complementing defective repair in Chinese hamster cells 2 (XRCC2), is a key factor and it contributes to the repair of DNA DSBs [18, 19]. Consequently, it has been hypothesized that XRCC2 may enhance tumor radioresistance [20]. Indeed, overexpression of XRCC2 has been shown to increase the radioresistance of glioblastoma multiforme, cervical cancer, and lung cancer cells [16, 21, 22]. In addition, we previously demonstrated that XRCC2 is overexpressed in colorectal cancer tissues and cell lines [23, 24]. However, it remains unclear whether XRCC2 overexpression increases radioresistance in LARC.

In this study, XRCC2 expression was detected in pretreatment biopsy LARC specimens and then was correlated with PRT responses and overall survival. In addition, the role of *XRCC2* in mediating the response of the SW480 cell line to IR was examined. In combination, these data were used to determine whether XRCC2 is a useful biomarker for guiding PRT in LARC.

## RESULTS

### XRCC2 expression was higher in specimens obtained from rectal cancer patients who underwent surgery without PRT and was also associated with TNM stage

**Figure 1:**
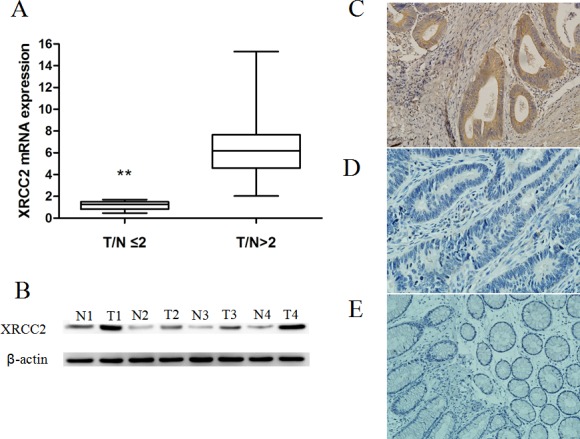
XRCC2 expression in the resected specimens that did not receive PRT **A.** Expression levels of *XRCC2* mRNA were higher in the 50 rectal cancer samples compared with the 50 corresponding normal colorectal mucosa tissue samples (Wilcoxon signed rank test, **P < 0.01). **B.** Expression of XRCC2 was detected by Western blotting and higher levels were present in the rectal cancer samples (T1-T4) compared with the matched adjacent non-tumor tissues (N1-N4). **C.**, **D.** Representative images of rectal cancer tissues that were positive **C.** and negative **D.** for XRCC2 expression in the immunohistochemical analysis performed (×200 magnification). **E.** A representative image of normal colorectal mucosa tissue that was negative for XRCC2 expression by immunohistochemistry (×200 magnification).

Levels of *XRCC2* mRNA were detected in 50 snap-frozen rectal cancer tissue samples and 50 matched, adjacent noncancerous tissue samples. The mRNA levels were significantly elevated (i.e., exhibited greater than a two-fold difference) in the rectal cancer tissues versus the adjacent noncancerous tissues (*P* < 0.01, Figure 1A). In subsequent Western blots, levels of XRCC2 protein were also higher in the rectal cancer samples than in the matched adjacent non-tumor tissues (Figure 1B). These results suggest that XRCC2 is upregulated in rectal cancer.

To further investigate the expression of XRCC2 *in situ*, paraffin-embedded rectal cancer tissue blocks (*n* = 100) were subjected to immunohistochemical analysis. Positive XRCC2 staining was only detected in 57/100 (57%) primary rectal cancer tissues (Figure 1C & 1D). In contrast, expression of XRCC2 was not detected in the adjacent non-tumor tissues (Figure 1E).

When XRCC2 expression and clinicopathological parameters were compared for the rectal patients of the present cohort, XRCC2 expression was found to significantly correlate with TNM stage (*P* < 0.05; Table 1). However, no correlation between XRCC2 expression and patient age, gender, lymph node metastasis, depth of invasion, or degree of differentiation was observed (*P* > 0.05; Table 1).

**Table 1:** Clinicopathological features and XRCC2 expression of rectal cancer patients who underwent surgery without PRT

Characteristics	Number	XRCC2 positive	XRCC2 negative	*P* value
Gender				0.223
Male	55	34	21	
Female	45	33	12	
Age (years)				0.840
≤60	38	25	13	
>60	62	42	20	
Tumor depth				0.750
T1/T2	20	14	6	
T3/T4	80	53	27	
Lymph node metastasis				0.721
Positive	48	33	15	
Negative	52	34	18	
Degree of differentiation				0.825
Well/moderate	83	56	27	
Poor/undifferentiated	17	11	6	
TNM stage				0.024
1	14	10	4	
2	31	15	16	
3	48	38	10	
4	7	6	1	

### Expression of XRCC2 in pretreatment biopsy tissue samples predicts postoperative histological tumor regression grade (TRG) and long-term prognosis in LARC patients who underwent surgery after PRT

**Figure 2:**
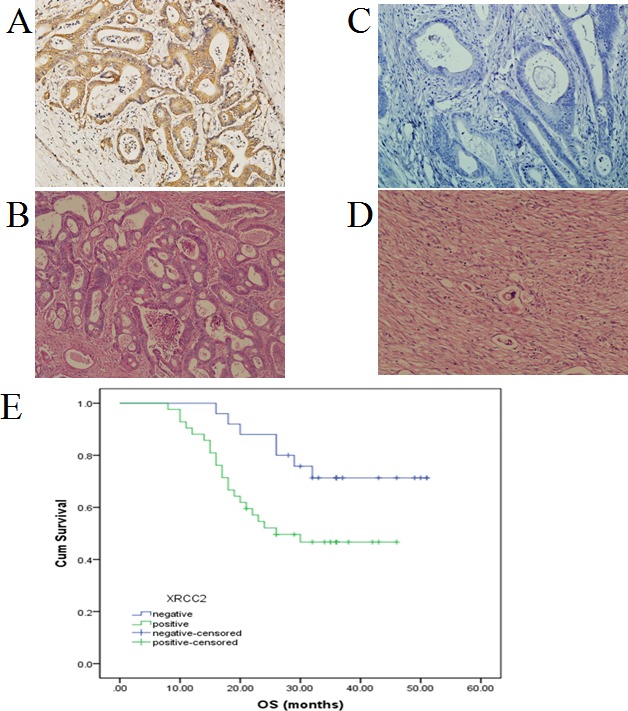
Decreased XRCC2 expression in pretreatment biopsy tissue samples of LARC patients who received PRT is associated with improved postoperative histological tumor regression and better long-term prognosis **A.** Positive XRCC2 expression in a representative biopsy specimens from a patient who exhibited resistance to PRT. **B.** Negative XRCC2 expression in a representative biopsy specimen from a patient who exhibited sensitivity to PRT. **C.** Positive XRCC2 expression in a representative biopsy specimen with poor tumor regression grade (TRG ≤ 2). **D.** Negative XRCC2 expression in a representative biopsy specimen with good tumor regression grade (TRG ≥ 3). **E.** Kaplan-Meier plot of overall survival. Survival was significantly improved in patients with XRCC2-negative tumors (blue line) than in patients with XRCC2-positive tumors (green line) (*P* < 0.01).

The associations between XRCC2 expression in pretreatment biopsy tissue samples and postoperative histological tumor regression and long-term prognosis were evaluated in 67 LARC patients who received PRT (Figure 2). Of these patients, 42/67 (62.7%) exhibited positive XRCC2 expression (Figure 2A) and 25/67 (37.3%) patients exhibited negative XRCC2 expression (Figure 2B). Following PRT, 40/67 (59.8%) cases showed a poor response (TRG ≤ 2) (Figure 2C), while in 27/67 (40.2%) cases, a good pathologic response was achieved (TRG ≥ 3) (Figure 2D). Of the latter, 18/27 (72.0%) cases were negative for XRCC2 expression, while 9/27 (21.5%) cases were positive for XRCC2 expression (Table 2). In addition, the overall 3-year survival rate for the XRCC2-negative group was significantly better than the XRCC2-positive group (71.2% vs. 46.7%, respectively; *P* < 0.01) (Figure 2E). Based on these results, it appears that XRCC2 is of clinical significance in the prognosis of patients with LARC who undergo surgery after PRT.

**Table 2:** Correlation between XRCC2 expression and tumor response to treatment according to TRG in locally advanced rectal cancer patients who underwent surgery after PRT

XRCC2 expression	Good response*	Poor response^#^	*P* value
Positive	9	33	0.0002
Negative	18	7	

* TRG≥3,

# TRG R≤2

### Validation of *XRCC2* knockdown in SW480 cells

**Figure 3:**
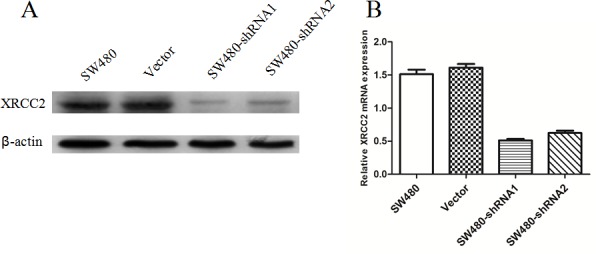
Successful knockdown of *XRCC2* in SW480 colorectal cancer cells **A.** Western blotting was used to detect *XRCC2* knockdown efficiency in untransfected SW480 cells, SW480 cells transfected with nonsilencing (vector) shRNAs, and SW480 cells transfected with shRNA1 and shRNA2. **B.** Lower expression of *XRCC2* mRNA was detected in *XRCC2* knockdown SW480 cells using quantitative real-time PCR.

Using lentivirus-mediated short hairpin RNAs (shRNAs) (XRCC2-sh1 and XRCC2-sh2), expression of *XRCC2* was knocked down in SW480 cells (Figure 3A & 3B). In particular, the SW480 cells that were infected with XRCC2-sh1 exhibited lower expression of XRCC2 compared with the controls, and these cells were used in subsequent experiments.

### Knockdown of *XRCC2* increased the radiosensitivity of human colorectal cancer cells

**Figure 4:**
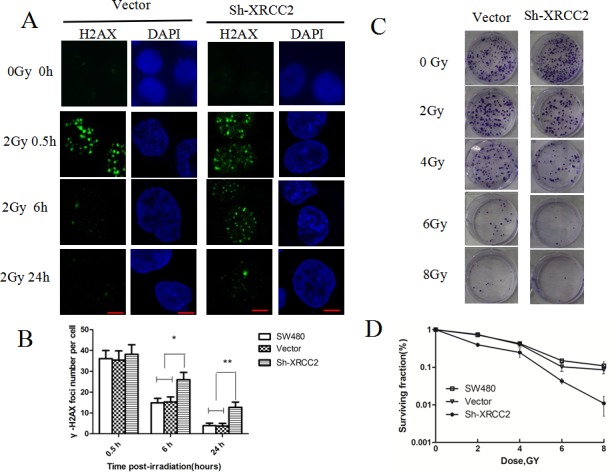
*XRCC2* knockdown cells exhibit impaired repair of radiation-induced DNA DSBs **A.** The levels of immunofluorescence that were observed in a phosphorylation of H2AX (γ-H2AX) assay. Images of γ-H2AX foci were obtained 0, 0.5, 6, and 24 h after 2 Gy of IR was applied to vector control cells and SW480 cells transduced with shRNA1. **B.** The average number of γ-H2AX foci per nucleus was calculated based on the number of γ-H2AX foci that were detected in more than 100 cells for each treatment group. The error bars represent the SD from three independent experiments. Statistical differences between the numbers of γ-H2AX foci in the control cells (SW480 and vector alone) and the shXRCC2 cells 6 h and 24 h after an IR treatment were calculated (*P < 0.05, **P < 0.01). **C.** Representative images of the clonogenic cell survival assays that were performed. IR treatment inhibited the colony-forming capacity of the cancer cells in a dose-dependent manner. Furthermore, sh-XRCC2 enhanced the tumor suppressive effect of the IR. **D.** The survival fraction curves for the SW480, vector, and sh-XRCC2 cells that were tested in clonogenic survival assays. Data shown are the mean ± SD of three independent experiments.

To examine the DSB repair efficiency of *XRCC2* knockdown cells compared to control cell lines, phosphorylation of H2AX (γ-H2AX) was assayed. In this assay, the persistence of γ-H2AX foci following IR reflects an impaired cellular capacity to repair DNA DSBs [25, 26]. Thus, γ-H2AX foci were assayed at different time points after the delivery of 2 Gy of IR. There was no difference in the number of γ-H2AX foci that were detected in the *XRCC2* knockdown cells compared to the control cells 0.5 h post-IR (Figure 4A). This was an expected result since IR induces DNA damage in both cell types. However, the γ-H2AX foci disappeared faster in the control cells at the 6 h and 24 h timepoints post-IR compared with the cells expressing sh-XRCC2 that were irradiated (Figure 4A & 4B). These results suggest that *XRCC2* may play a role in enhancing the capacity of cells to repair radiation-induced DNA DSBs.

To further support this DNA repair role of XRCC2, we performed a clonogenic cell survival assay in the three cell lines after IR. Following the application of IR to the sh-XRCC2, vector, and SW480 cell lines, a dose-dependent decrease in the survival of all three cell lines was observed. However, the sh-XRCC2 cells formed fewer colonies following the IR treatment compared to the controls (Figure 4C & 4D). Taken together, these results confirm that *XRCC2* expression contributes to radioresistance in colorectal cancer cells.

### Knockdown of *XRCC2* enhanced radiation-induced apoptosis

**Figure 5:**
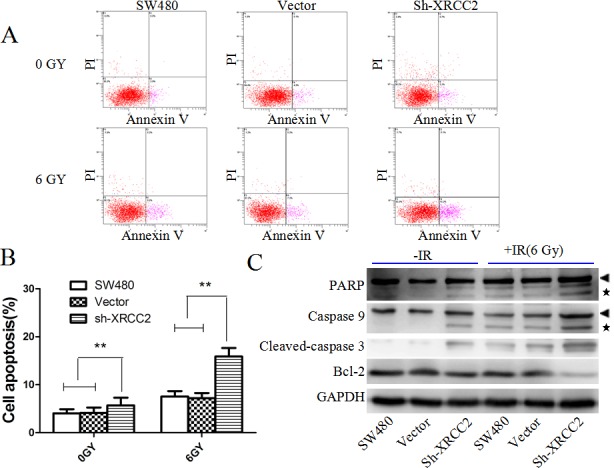
*XRCC2* knockdown significantly increases cell apoptosis that is induced by IR treatment **A.** Levels of apoptosis in the *XRCC2* knockdown cells versus the control cells (SW480 and vector alone) that were treated with 6 Gy of IR for 24 h. The cells were stained with Annexin V and PI. **B.** The percentage of cells that underwent apoptosis following IR treatment were quantitated and the data are expressed as the mean ± SD from three independent experiments (*P < 0.05, **P < 0.01). **C.** Detection of PARP, caspase-9, cleaved caspase-3, and Bcl-2 protein levels by Western blotting. Arrow heads and stars represent the pro-forms and the cleaved forms of PARP and caspase 9, respectively. Detection of GAPDH was used as a loading control.

In our previous study, XRCC2 overexpression was found to inhibit colorectal cancer cell apoptosis [23]. To determine whether XRCC2-mediated radioresistance in colorectal cancer cells is also due to inhibition of apoptosis, cell apoptosis was detected by flow cytometry. As shown in Figures 5A and 5B, the vector and SW480 cells exhibited a significant reduction in radiation-induced apoptosis compared with the *XRCC2* knockdown cells (*P* < 0.01). To investigate the possible mechanisms for this observed increase in radiosensitivity, biochemical markers of apoptosis were detected. These markers included: poly adenosine diphosphate ribose polymerase (PARP), cleaved caspase-9, cleaved caspase-3, and anti-apoptosis (Bcl-2) [27,28]. Higher levels of cleaved PARP, cleaved caspase-9, and cleaved caspase-3 were detected in the sh-XRCC2 cells compared to the vector and SW480 cells 24 h after 6 Gy of IR was applied to the cells (Figure 5C). In contrast, a significant decrease in the levels of Bcl-2 were detected in the sh-XRCC2 cells following IR (Figure 5C). Taken together, these findings indicate that XRCC2 overexpression may protect LARC cells from IR-induced apoptosis.

### *XRCC2* knockdown increased radiation-induced G2/M arrest and p-Chk2 activation

**Figure 6:**
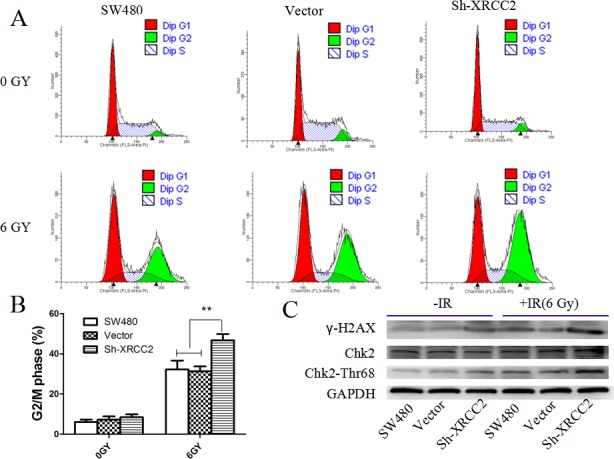
*XRCC2* knockdown increases cell cycle arrest in the G2 phase and activates p-Chk2 after IR treatment **A.** Flow cytometry analysis of the proportion of the cells indicated in each stage of the cell cycle (G1, G2, and S) after the IR treatment. **B.** The percentage of cells in the G2/M phase of the cell cycle were quantitated and expressed as the mean ± SEM from three independent experiments (*P < 0.01). **C.** Expression levels of the γ-H2AX, phosphorylated Chk2-Thr68, and Chk2 proteins that were detected by Western blotting in the SW480, vector, and Sh-XRCC2 cell lines. Expression levels of phosphorylated Chk2-Thr68 and -H2AX were higher in the sh-XRCC2 cells compared to the controls after IR. Detection of GAPDH was used as a loading control.

Cells arrested at the G2/M point of the cell cycle are generally more sensitive to radiation than cells that are arrested in other phases of the cell cycle [29]. To determine the cell cycle progression of colorectal cancer cells after exposure to IR, exponentially growing cancer cells were exposed to 6 Gy of IR and then were analyzed for DNA content 24 h later by flow cytometry. *XRCC2* knockdown was found to significantly disturb cell cycle progression (Figure 6A & 6B). Specifically, there was a dramatic increase in the percentage of sh-XRCC2 cells in the G2/M phase compared with the control cells (Figure 6B), thereby indicating that downregulation of *XRCC2* in LARC cells leads to an arrest of the cell cycle in the G2/M phase. To confirm these findings, changes in Chk2, a key effector protein of the G2/M checkpoint [30,31], were monitored. As shown in Figure 6C, higher levels Chk2-Thr68 phosphorylation were detected in sh-XRCC2 cells compared to control cells 24 h after the cells were treated with 6 Gy of IR. Therefore, knockdown of *XRCC2* in colorectal cancer cells may activate Chk2-Thr68, thereby leading to G2/M cell cycle arrest and improved radiosensitivity.

## DISCUSSION

Lower levels of XRCC2 expression have been found in breast cancer tissues [32], while higher levels of XRCC2 expression have been detected in several other cancers [16, 23, 33]. It is hypothesized that XRCC2 expression is involved in either the initiation or progression of tumorigenesis [23, 34]. In the present study, XRCC2 expression was detected in freshly frozen rectal cancer specimens, and both mRNA and protein levels of XRCC2 were found to be higher in rectal cancer tissues than in matched adjacent noncancerous tissues. In the paraffin specimens obtained from rectal cancer patients, positive XRCC2 expression was found to be associated with TNM stage (*P* = 0.024). These results suggest that overexpression of XRCC2 may promote the invasive behavior of rectal cancer. Therefore, to our knowledge, we have provided the first clinical evidence that XRCC2 may play an important role in the progression of rectal cancer.

PRT has become a standard treatment for LARC, however, not all patients are radiosensitive. TRG has been used to capture the early response of LARC to PRT, since this score has previously been used as a prognostic factor in rectal cancer [35-37]. In the present study, negative XRCC2 expression was associated with a higher TRG score after PRT. Thus, XRCC2 appears to be an independent biomarker for predicting how well rectal cancer will respond to PRT. In addition, 3-year overall and disease-free survival rates have previously been used to evaluate the long-term effects of therapies for colon cancer patients [38]. In the present cohort, LARC patients with high levels of XRCC2 expression were found to have poorer progression-free survival. Therefore, we propose that XRCC2 overexpression may be predictive of tumor resistance to PRT.

Due to the observed association between XRCC2 and poor PRT results in LARC patients, we sought to determine the mechanisms that mediate XRCC2-induced radioresistance. Cell cycle phase is one of the most important determinants of radiosensitivity, and cells arrested in the G2 phase are generally more sensitive to radiation than cells that are arrested in other phases of the cell cycle [39, 40]. Correspondingly, when *XRCC2* was knocked down in the SW480 cells, a greater number of cells arrested in the G2/M phase after exposure to the IR. These results suggest that cell cycle arrest in the G2/M phase is enhanced in the tumor cells of XRCC2-negative LARC patients, and this leads to improved radiosensitivity.

Classically, it has been proposed that HRR is a relatively slow process and it only occurs during the G2/M phase [41]. When we examined whether *XRCC2* knockdown suppressed the repair of DNA DSBs, it was observed that the number of γ-H2AX foci, which are used to measure DNA DSBs, were higher in *XRCC2* knockdown cells than in the control cells, especially 6 h and 24 h after IR treatment. These results indicate that inefficient HRR occurred in the irradiated cells that had *XRCC2* knocked down. Furthermore, *XRCC2* knockdown exacerbated the G2/M arrest after the IR treatment, and a majority of the damaged cells in the G2/M phase could not be repaired via homologous recombination.

Disruption of the signaling pathways that lead to apoptosis is a key mechanism by which cancer cells become radioresistant [42]. In the present study, *XRCC2* inhibition significantly increased the extent of IR-induced apoptosis that was detected, thereby supporting the hypothesis that XRCC2 plays a role in apoptosis evasion. To further explore the mechanism by which XRCC2 mediates radioresistance, levels of PARP, caspase-9, and cleaved caspase-3 were detected. In the *XRCC2* knockdown cells that were treated with IR, significantly higher levels of cleaved PARP, caspase-9, and caspase-3 were detected, along with lower levels of Bcl-2, compared to control cells. Thus, XRCC2 may confer radioresistance through anti-apoptotic pathways.

IR induces Ataxia-telangiectasia mutated (ATM)- and Chk2-dependent checkpoint signaling, and this induces apoptosis in sublethally damaged cancer cells [43,44]. Conversely, reduced Chk2 phosphorylation may facilitate the escape of cancer cells from apoptosis, thereby leading to radioresistance [45,46]. Increased levels of phosphorylated Chk2 were detected in the *XRCC2* knockdown cells of the present study compared to the controls cells after IR, and these results suggest that in XRCC2-negative LARC patients, activation of Chk2-dependent signaling may improve tumor cell radiosensitivity.

In conclusion, the results of this study demonstrate that XRCC2 is upregulated in rectal cancer compared to adjacent normal rectal tissues and this may promote rectal cancer progression. Furthermore, a functional relationship between XRCC2 overexpression and reduced radiosensitivity was observed *in vivo*, and this was consistent with the observations that *XRCC2*-negative tumor cells exhibited an increase in G2/M cell cycle phase arrest and apoptosis-related cell death. In combination, these results suggest that detection of XRCC2 expression in pretreatment biopsy specimens has the potential to be a predictive factor for PRT response in LARC patients. These results remain to be confirmed in a strict, randomized, and controlled clinical trial, although the ability to reduce XRCC2 expression or activity may represent a promising therapeutic option for radioresistant LARC that is not well-controlled by traditional radiotherapy.

## MATERIALS AND METHODS

### Patients and the clinical database

Resection specimens were collected from patients with primary rectal cancer who underwent gastrointestinal surgery without PRT between January 2013 and July 2014 at The First Affiliated Hospital of Sun Yat-sen University. All excised tissues were immediately frozen in liquid nitrogen and then were stored at −80°C. In addition, 67 patients clinically diagnosed with LARC (T3-4 and/or N0-2 lesions) were recruited between January 2010 and December 2012. Biopsy tissue samples were obtained before these patients received PRT involving a total dose of 45.0-50.4 Gy of IR to the pelvic region in 25-28 fractions over a period of five weeks. Radical surgery (low anterior resection or abdominoperineal resection) was performed 2-3 weeks after completing PRT at The First Affiliated Hospital of Sun Yat-sen University or at the Huaihe Hospital of Henan University.

### Immunohistochemistry and tumor regression grading (TRG)

Immunohistochemical analyses of rectal cancer tissue samples were performed to detect the expression and subcellular localization of XRCC2, according to our previous experiment protocol [23]. Pathological tumor response to PRT was evaluated according to hematoxylin and eosin-stained slides using the TRG system as previously described [47]. The characteristics of each grade were as follows: TRG 0, no regression; TRG 1, dominant tumor mass with obvious fibrosis in ≤ 25% of the tumor mass; TRG 2, dominant tumor mass with obvious fibrosis in 26-50% of the tumor mass; TRG 3, dominant fibrosis outgrowing the tumor mass; and TRG 4, no viable tumor cells (only a fibrotic mass). Immunohistochemical and TRG evaluations were performed by two pathologists that were blinded to the clinical and pathological characteristics associated with the specimens.

### Vector and retroviral infection

The colorectal cancer cell line, SW480, was obtained from American Type Culture Collection (Manassas, VA, USA). The cells were cultured in RPMI 1640 medium (Life Technologies, Carlsbad, CA, USA) that was supplemented with 10% fetal bovine serum (HyClone, Logan, UT, USA) and 1% penicillin/streptomycin. All cells were maintained in a humidified atmosphere at 37°C with 5% CO_2_.

Several lentiviral-based plasmids, each containing a short hairpin RNA (shRNA) designed to target human *XRCC2* were purchased from RiboBio Co., Ltd (Guangzhou, China). Nonsilencing (vector) shRNAs were used as negative controls. The infection of SW480 cells with human *XRCC2*-targeted shRNAs was performed as previously described [23, 48]. Briefly, stable clones were generated by transfecting SW480 cells in 6-well dishes with 2 μg of each shRNA plasmid. Forty-eight hours later, antibiotic selection with 2.0 μg/mL puromycin was initiated. After 10 d, multiple clones from the same transfection were pooled and were grown under puromycin selection. The knockdown efficiency of XRCC2 was examined by quantitative RT-PCR and western blots.

### Western blotting

Western blotting was performed as previously described [49]. Briefly, equal amounts of lysates were resolved with SDS-PAGE and were transferred onto PVDF membranes. Membranes were incubated with primary antibodies followed by horseradish peroxidase (HRP)-conjugated secondary antibodies. Signal amplification and detection were achieved by exposing the membrane to enhanced chemiluminescence reagent (GE Healthcare, Buckinghamshire, UK), followed by visualization using the Storm imaging system (Amersham Biosciences, Piscataway, NJ, USA). The following primary antibodies were used: γ-H2AX (1/1000; Cell Signaling Technology, Beverly, MA, USA), Chk2 (1/500; Cell Signaling Technology), Phospho-Chk2 (Thr68) (1/1000; Cell Signaling Technology), XRCC2 (1:1500; Abcam), Caspase-9 (1/500; Proteintech, Chicago, IL, USA), Caspase-3 (1/500; Proteintech), PARP (1/500; Proteintech), and BCL-2 (1/500; Santa Cruz Biotechnology). Detection of GADPH (1/10000; Cell Signaling Technology) was used as a loading control. Bound antibodies were visualized with peroxidase-linked secondary antibodies (anti-rabbit antibody: 1/10000; Cell Signaling Technology and anti-mouse antibody: 1/5000; Sigma-Aldrich, St. Louis, MO, USA).

### Quantitative real-time PCR (qRT-PCR)

Total RNA was extracted and qRT-PCR was performed as described in our previous study [23]. All experiments were performed at least in triplicate.

### Immunofluorescence

A previously reported immunofluorescence protocol was used [16, 50], with some modifications. Cells were irradiated with 2 Gy of IR after they were attached to chamber slides (Nest, Wuxi, China) in 24-well plates. At various timepoints following exposure to IR (0, 0.5, 6 and 24 h), the cells were washed three times in PBS and then were fixed in 4% paraformaldehyde at room temperature. After 15 min, the cells were permeabi­lized in 0.2% Triton X-100 for 15 min, then were washed three times in PBS and were blocked in 10% goat serum for 1 h. The coverslips were incubated with anti-γ-H2AX monoclonal antibody (Cell Signaling Technology) diluted 1:100 in 1% BSA/PBS/0.1% Tween overnight at 4°C. After being washed three times with PBS, the cells were incubated with DyLight 488 AffiniPure Goat Anti-Mouse IgG (Abbkine, Redlands, CA, USA) at room temperature in the dark. After 1 h, the cells were washed with PBS three times before being counterstained with 1 μg/ml 4′,6-diamidino-2-phenylindole (DAPI) for 3 min in the dark. Finally, the slides were mounted with an antifading reagent and were examined with a confocal microscope (Zeiss, Germany). In each sample, the number of γ-H2AX foci per nucleus were counted using an automated foci counter under a high-power field, and an average of 100 nuclei were analyzed.

### Clonogenic cell survival assay

Cellular sensitivity to radiation was determined by loss of colony-forming ability as described previously [51]. Briefly, exponentially growing cells were seeded into 6-well plates (500 cells/well). After 24 h, the cells were exposed to varying doses of IR (0, 2, 4, 6, and 8 Gy) and then were kept in an incubator at 37°C with 5% CO_2_ for 10-14 d. The colonies were fixed with methanol for 30 min and then were stained with crystal violet. Only colonies with more than 50 cells were counted manually. The surviving fraction was calculated using GraphPad Prism 5.0 software (GraphPad Software, Inc., San Diego, CA, USA) based on a muti-target/single-hit model (y = 1 − (1 − exp(−k*x)) ^ N). Experiments were performed in triplicate and were repeated three times.

### Flow cytometry analysis of cell cycle and apoptosis

Cultured cells were harvested 24 h after receiving 6 Gy of IR and then cell cycle progression and apoptosis were analyzed by flow cytometry. For cell cycle analysis, the cells were trypsinized and washed in PBS for 5 min prior to collection by centrifugation at 1500 rpm. The cells were then fixed with ice-cold 70% ethanol at −20°C overnight. The fixed cells were subsequently stained with 20 mg/mL propidium iodide (PI) staining buffer (containing 1% Triton X-100 and 100 mg/mL RNase A) for 30 min. DNA content was assessed using a FACSCalibur unit (Becton Dickinson, Franklin Lakes, NJ, USA) equipped with ModFit LT v2.0 software. For the apoptosis assay, the cells were harvested by trypsinization and were washed with PBS. The cells were resuspended in 1× binding buffer at a concentration of 3 × 10^6^ cells/mL. After staining the cells with fluorescein isothiocyanate (FITC) Annexin V and PI, the cells were analyzed using an Epics Profile II flow cytometer (Beckman Coulter, Fullerton, CA, USA) and Multicycle software (Phoenix Flow Systems, San Diego, CA, USA). All of the experiments were repeated at least three times.

### Statistical analysis

The paired-samples Wilcoxon signed rank test was used to compare the expression of XRCC2 between tumor and adjacent normal tissues. A two-fold difference was considered the cut-off point for defining high versus low levels of expression. The correlation between XRCC2 expression and clinicopathological features was examined using Pearson's Chi-squared test. Survival rates were calculated using the Kaplan-Meier method. One-way analysis of variance (ANOVA) was used to compare quantitative data among the different cell groups. All values are expressed as the mean ± standard deviation (SD). A *P*-value of less than 0.05 was considered to be statistically significant. The statistical software package SPSS (version 17.0; IBM, Armonk, NY, USA) was employed for all analyses.

## References

[1] Xu AG, Yu ZJ, Jiang B, Wang XY, Zhong XH, Liu JH, Lou QY, Gan AH (2010). Colorectal cancer in Guangdong Province of China: a demographic and anatomic survey. World J Gastroenterol.

[2] de Campos-Lobato LF, Stocchi L, da Luz Moreira A, Geisler D, Dietz DW, Lavery IC, Fazio VW, Kalady MF (2011). Pathologic complete response after neoadjuvant treatment for rectal cancer decreases distant recurrence and could eradicate local recurrence. Ann Surg Oncol.

[3] Huh JW, Kim HR, Kim YJ (2013). Clinical prediction of pathological complete response after preoperative chemoradiotherapy for rectal cancer. Dis Colon Rectum.

[4] Baker B, Salameh H, Al-Salman M, Daoud F (2012). How does preoperative radiotherapy affect the rate of sphincter-sparing surgery in rectal cancer?. Surg Oncol.

[5] Minsky BD (2011). Progress in the treatment of locally advanced clinically resectable rectal cancer. Clin Colorectal Cancer.

[6] Sauer R, Liersch T, Merkel S, Fietkau R, Hohenberger W, Hess C, Becker H, Raab HR, Villanueva MT, Witzigmann H, Wittekind C, Beissbarth T, Rödel C (2012). Preoperative versus postoperative chemoradiotherapy for locally advanced rectal cancer: results of the German CAO/ARO/AIO-94 randomized phase III trial after a median follow-up of 11 years. J Clin Oncol.

[7] Martin ST, Heneghan HM, Winter DC (2012). Systematic review and meta-analysis of outcomes following pathological complete response to neoadjuvant chemoradiotherapy for rectal cancer. Br J Surg.

[8] Qiu J, Yang G, Shen Z, Xie Y, Wang L (2013). hPEBP4 as a predic tive marker for the pathological response of rectal cancer to preoperative radiotherapy. Int J Colorectal Dis.

[9] Sakai K, Kazama S, Nagai Y, Murono K, Tanaka T, Ishihara S, Sunami E, Tomida S, Nishio K, Watanabe T (2014). Chemoradiation provides a physiological selective pressure that increases the expansion of aberrant TP53 tumor variants in residual rectal cancerous regions. Oncotarget.

[10] Moussata D, Amara S, Siddeek B, Decaussin M, Hehlgans S, Paul-Bellon R, Mornex F, Gerard JP, Romestaing P, Rödel F, Flourie B, Benahmed M, Mauduit C (2012). XIAP as a radioresistance factor and prognostic marker for radiotherapy in human rectal adenocarcinoma. Am J Pathol.

[11] Kuremsky JG, Tepper JE, McLeod HL (2009). Biomarkers for response to neoadjuvant chemoradiation for rectal cancer. Int J Radiat Oncol Biol Phys.

[12] Perez RO (2011). Predicting response to neoadjuvant treatment for rectal cancer: a step toward individualized medicine. Dis Colon Rectum.

[13] Chua ML, Rothkamm K (2013). Biomarkers of radiation exposure: can they predict normal tissue radiosensitivity?. Clin Oncol (R Coll Radiol).

[14] Scott SP, Pandita TK (2006). The cellular control of DNA double-strand breaks. J. Cell Biochem.

[15] Liu H, Sun X, Zhang S, Ge W, Zhu Y, Zhang J, Zheng S (2011). The dominant negative mutant Artemis enhances tumor cell radiosensitivity. Radiother Oncol.

[16] Zheng Z, Ng WL, Zhang X, Olson JJ, Hao C, Curran WJ, Wang Y (2012). RNAi-Mediated Targeting of Noncoding and Coding Sequences in DNA Repair Gene Messages Efficiently Radiosensitizes Human Tumor Cells. Cancer Res.

[17] Mladenov E, Magin S, Soni A, Iliakis G (2013). DNA double-strand break repair as determinant of cellular radiosensitivity to killing and target in radiation therapy. Front Oncol.

[18] Tambini CE, Spink KG, Ross CJ, Hill MA, Thacker J (2010). The importance of XRCC2 in RAD51-related DNA damage repair. DNA Repair.

[19] Johnson RD, Liu N, Jasin M (1999). Mammalian XRCC2 promotes the repair of DNA double-strand breaks by homologous recombination. Nature.

[20] Haines JW, Coster MR, Adam J, Cheeseman M, Ainsbury EA, Thacker J, Bouffler SD (2010). Xrcc2 Modulates Spontaneous and Radiation-Induced Tumorigenesis in Apcmin/+ Mice. Mol Cancer Res.

[21] Liu Y, Shete S, Wang LE, El-Zein R, Etzel CJ, Liang FW, Armstrong G, Tsavachidis S, Gilbert MR, Aldape KD, Xing J, Wu X, Wei Q, Bondy ML (2010). γ-Radiation sensitivity and polymorphisms in RAD51L1 modulate glioma risk. Carcinogenesis.

[22] Paulíková S, Chmelařová M, Petera J, Palička V, Paulík A (2013). Hypermethylation of RAD51L3 and XRCC2 genes to predict late toxicity in chemoradiotherapy-treated cervical cancer patients. Folia Biol (Praha).

[23] Xu K, Song X, Chen Z, Qin C, He Y, Zhan W (2014). XRCC2 promotes colorectal cancer cell growth, regulates cell cycle progression, and apoptosis. Medicine (Baltimore).

[24] Xu K, Song X, Chen Z, Qin C, He Y (2014). XRCC2 rs3218536 polymorphism decreases the sensitivity of colorectal cancer cells to poly(ADP-ribose) polymerase 1 inhibitor. Oncol Lett.

[25] Redon CE, Nakamura AJ, Gouliaeva K, Rahman A, Blakely WF, Bonner WM (2010). The Use of Gamma-H2AX as a biodosimeter for total-body radiation exposure in non-human primates. PLoS One.

[26] Rothkamm K, Löbrich M (2003). Evidence for a lack of DNA double-strand break repair in human cells exposed to very low x-ray doses. Proc Natl Acad Sci U S A.

[27] Nicholson DW (1999). Caspase structure, proteolytic substrates, and function during apoptotic cell death. Cell Death Differ.

[28] Antonsson B, Martinou JC (2000). The Bcl-2 protein family. Exp Cell Res.

[29] Nagasawa H, Keng P, Harley R, Dahlberg W, Little JB (1994). Relationship between gamma-ray-induced G2/M delay and cellular radiosensitivity. Int J Radiat Biol.

[30] Smits VA, Medema RH (2001). Checking out the G(2)/M transition. Biochim Biophys Acta.

[31] Rainey MD, Black EJ, Zachos G, Gillespie DA (2008). Chk2 is required for optimal mitotic delay in response to irradiation induced DNA damage incurred in G2 phase. Oncogene.

[32] Bashir N, Sana S, Mahjabeen I, Kayani MA (2014). Association of reduced XRCC2 expression with lymph node metastasis in breast cancer tissues. Fam Cancer.

[33] Gok I, Baday M, Cetinkunar S, Kilic K, Bilgin BC (2014). Polymorphisms in DNA repair genes XRCC2 and XRCC3 risk of gastric cancer in Turkey. Bosn J Basic Med Sci.

[34] Krupa R, Sliwinski T, Wisniewska-Jarosinska M, Chojnacki J, Wasylecka M, Dziki L, Morawiec J, Blasiak J (2011). Polymorphisms in RAD51, XRCC2 and XRCC3 genes of the homologous recombination repair in colorectal cancer—a case controlstudy. Mol Biol Rep.

[35] Yao YF, Du CZ, Chen N, Chen P, Gu J (2014). Expression of HER-2 in rectal cancers treated with preoperative radiotherapy: a potential biomarker predictive of metastasis. Dis Colon Rectum.

[36] Mace AG, Pai RK, Stocchi L, Kalady MF (2015). American joint committee on cancer and college of american pathologists regression grade: a new prognostic factor in rectal cancer. Dis Colon Rectum.

[37] Maas M, Nelemans PJ, Valentini V, Das P, Rödel C, Kuo LJ, Calvo FA, García-Aguilar J, Glynne-Jones R, Haustermans K, Mohiuddin M, Pucciarelli S, Small W, Suárez J, Theodoropoulos G, Biondo S, Beets-Tan RG, Beets GL (2010). Long-term outcome in patients with a pathological complete response after chemoradiation for rectal cancer: a pooled analysis of individual patient data. Lancet Oncol.

[38] Sargent D, Shi Q, Yothers G, Van Cutsem E, Cassidy J, Saltz L, Wolmark N, Bot B, Grothey A, Buyse M, de Gramont A, Adjuvant Colon Cancer End-points (ACCENT)Group (2011). Two or three year disease-free survival (DFS) as a primary end-point in stage III adjuvant colon cancer trials with fluoropyrimidines with or without oxaliplatin or irinotecan: data from 12,676 patients from MOSAIC, X-ACT, PETACC-3, C-06, C-07 and C89803. Eur J Cancer.

[39] Pawlik TM, Keyomarsi K (2004). Role of cell cycle in mediating sensitivity to radiotherapy. Int J Radiat Oncol Biol Phys.

[40] Yan Y, Hein AL, Etekpo A, Burchett KM, Lin C, Enke CA, Batra SK, Cowan KH, Ouellette MM (2014). Inhibition of RAC1 GTPase sensitizes pancreatic cancer cells toγ-irradiation. Oncotarget.

[41] Takata M, Sasaki MS, Sonoda E, Morrison C, Hashimoto M, Utsumi H, Yamaguchi-Iwai Y, Shinohara A, Takeda S (1998). Homologous recombination and non-homologous end-joining pathways of DNA double-strand break. EMBO J.

[42] Dai Y, Liu M, Tang W, DeSano J, Burstein E, Davis M, Pienta K, Lawrence T, Xu L (2008). Molecularly targeted radiosensitization of human prostate cancer by modulating inhibitor of apoptosis. Clin Cancer Res.

[43] Wang WJ, Wu SP, Liu JB, Shi YS, Huang X, Zhang QB, Yao KT (2013). MYC regulation of CHK1 and CHK2 promotes radioresistance in a stem cell-like population of nasopharyngeal carcinoma cells. Cancer Res.

[44] Gogineni VR, Nalla AK, Gupta R, Dinh DH, Klopfenstein JD, Rao JS (2011). Chk2-mediated G2/M cell cycle arrest maintains radiation resistance in malignant meningioma cells. Cancer Lett.

[45] Squatrito M, Brennan CW, Helmy K, Huse JT, Petrini JH, Holland EC (2010). Loss of ATM/Chk2/p53 pathway components accelerates tumor development and contributes to radiation resistance in gliomas. Cancer Cell.

[46] Lin HY, Hung SK, Lee MS, Chiou WY, Huang TT, Tseng CE, Shih LY, Lin RI, Lin JM, Lai YH, Chang CB, Hsu FC, Chen LC, Tsai SJ, Su YC, Li SC, Lai HC, Hsu WL, Liu DW, Tai CK, Wu SF, Chan MW (2015). DNA methylome analysis identifies epigenetic silencing of FHIT as a determining factor for radiosensitivity in oral cancer: an outcome-predicting and treatment-implicating study. Oncotarget.

[47] Dworak O, Keilholz L, Hoffmann A (1997). Pathological features of rectal cancer after preoperative radiochemotherapy. Int J Colorectal Dis.

[48] Vilar E, Bartnik CM, Stenzel SL, Raskin L, Ahn J, Moreno V, Mukherjee B, Iniesta MD, Morgan MA, Rennert G, Gruber SB (2011). MRE11 deficiency increases sensitivity to poly (ADP-ribose) polymerase inhibition in microsatellite unstable colorectal cancers. Cancer Res.

[49] Pérès EA, Gérault AN, Valable S, Roussel S, Toutain J, Divoux D, Guillamo JS, Sanson M, Bernaudin M, Petit E (2015). Silencing erythropoietin receptor on glioma cells reinforces efficacy of temozolomide and X-rays through senescence and mitotic catastrophe. Oncotarget.

[50] Liu WL, Gao M, Tzen KY, Tsai CL, Hsu FM, Cheng AL, Cheng JC (2014). Targeting Phosphatidylinositide3-Kinase/Akt pathway by BKM120 for radiosensitization in hepatocellular carcinoma. Oncotarget.

[51] Kuo PL, Hsu YL, Cho CY (2006). Plumbagin induces G2-M arrest and autophagy by inhibiting the AKT/mammalian target of rapamycin pathway in breast cancer cells. Mol Cancer Ther.

